# Telomere Length Calibration from qPCR Measurement: Limitations of Current Method

**DOI:** 10.3390/cells7110183

**Published:** 2018-10-24

**Authors:** Youjin Wang, Sharon A. Savage, Rotana Alsaggaf, Geraldine Aubert, Casey L. Dagnall, Stephen R. Spellman, Stephanie J. Lee, Belynda Hicks, Kristine Jones, Hormuzd A. Katki, Shahinaz M. Gadalla

**Affiliations:** 1 Clinical Genetics Branch, Division of Cancer Epidemiology and Genetics, National Cancer Institute, National Institutes of Health, Bethesda, MD 20892, USA; youjin.wang@nih.gov (Y.W.); savagesh@mail.nih.gov (S.A.S.); rotana.alsaggaf@nih.gov (R.A.); 2 Terry Fox Laboratory, British Columbia Cancer Agency, Vancouver, BC V5Z 1L3, Canada; gaubert@bccrc.ca; 3 Repeat Diagnostics Inc., North Vancouver, BC V7M 1A5, Canada; 4 Cancer Genomics Research Laboratory, Division of Cancer Epidemiology and Genetics, National Cancer Institute, National Institutes of Health, Bethesda, MD 20892, USA; dagnallc@mail.nih.gov (C.L.D.); hicksbel@mail.nih.gov (B.H.); kristine.jones@nih.gov (K.J.); 5 Leidos Biomedical Research, Inc., Frederick National Laboratory for Cancer Research, Frederick, MD 21701, USA; 6 Center for International Blood and Marrow Transplant Research, Minneapolis, MN 55401, USA; sspellma@nmdp.org; 7 Center for International Blood and Marrow Transplant Research, Medical College of Wisconsin, Milwaukee, WI 53226, USA; sjlee@fredhutch.org; 8 Clinical Research Division, Fred Hutchinson Cancer Research Center, Seattle, WA 98109, USA; 9 Biostatistics Branch, Division of Cancer Epidemiology and Genetics, National Cancer Institute, National Institutes of Health, Bethesda, MD 20892, USA; katkih@mail.nih.gov

**Keywords:** telomere length, qPCR, flow FISH, agreement

## Abstract

Telomere length (TL) comparisons from different methods are challenging due to differences in laboratory techniques and data configuration. This study aimed to assess the validity of converting the quantitative polymerase chain reaction (qPCR) telomere/single copy gene (T/S) ratio to TL in kilobases (kb). We developed a linear regression equation to predict TL from qPCR T/S using flow cytometry with fluorescence in situ hybridization (flow FISH) TL data from 181 healthy donors (age range = 19–53) from the National Marrow Donor Program (NMDP) biorepository. TL measurements by qPCR and flow FISH were modestly correlated (*R*^2^ = 0.56, *p* < 0.0001). In Bland-Altman analyses, individuals with the shortest (≤10th percentile) or longest (≥90th) flow FISH TL had an over- or under-estimated qPCR TL (bias = 0.89 and −0.77 kb, respectively). Comparisons of calculated TL from the NMDP samples and 1810 age- and sex-matched individuals from the National Health and Nutrition Examination Survey showed significant differences (median = 7.1 *versus* 5.8 kb, respectively, *p* < 0.0001). Differences in annual TL attrition were also noted (31 *versus* 13 bp/year, respectively, *p* = 0.02). Our results demonstrate that TL calculated in kb from qPCR T/S may yield biased estimates for individuals with the shortest or longest TL, those often of high clinical interest. We also showed that calculated TL in kb from qPCR data are not comparable across populations and therefore are not necessarily useful.

## 1. Introduction 

Telomeres consist of hexanucleotide (TTAGGG)_n_ repeats at the end of chromosomes capped by a protein complex [1]. Telomeres protect the end of the chromosomes from fusions and from being recognized as DNA breaks [2]. They shorten with each cell division, due to the incomplete replication of terminal DNA by conventional DNA polymerases [3]. Critically short telomeres can trigger genomic instability [4]. 

Inherited mutations in telomere-biology genes are recognized as the underlying biological mechanism in the telomere-biology disorders; dyskeratosis congenita (DC), and subsets of patients with severe aplastic anemia (SAA) or idiopathic pulmonary fibrosis (IPF) [5]. Such patients usually have a telomere length (TL) <10th percentile-for-age by flow cytometry with fluorescence in situ hybridization (flow FISH) with DC patients having the shortest TL (<1st percentile-for-age) [6,7,8]. In the general population, TL has been linked to a number of age-related diseases such as cancer [9,10,11] and heart disease [12,13]. Recent studies also suggest a role for TL in optimal donor selection for hematopoietic cell transplantation (HCT) in patients with SAA [14,15,16]. 

Several TL measurement methods exist; the most commonly used are Southern blot, flow FISH, and quantitative polymerase chain reaction (qPCR). In Southern blots, TL is determined in kilobases (kb) based on the terminal restriction fragment (TRF) length after DNA digestion with restriction endonucleases [17]. This method requires a large amount of DNA (~3 μg), which limits its applicability in many studies. In addition, TRF TL measurement includes the subtelomeric region, which may result in a TL overestimation. The flow FISH assay uses a telomere specific fluorescently labeled peptide nucleic acid (PNA) probe, and calculates the TL of distinct leukocyte subsets in kb based on the TRF calibration of a reference sample [18]. The qPCR technique of TL measurement estimates the relative ratio of the telomeric repeat amplified sequence to that of a single copy gene (T/S) [19]. It has emerged as a useful tool to measure TL in large epidemiology studies because of its high throughput capabilities and low DNA input requirement. 

The absence of absolute quantification of TL with the qPCR assay prompted the use of conversion equations to determine the kb of TL in several studies [20,21,22,23]. Evaluating the validity of such an approach is particularly important in the context of its use in studies of clinical or public health impact. Here, we compared TL in kb from flow FISH data and that calculated using conversion equations from qPCR T/S measurements on the same samples. We also evaluated whether the use of such an approach can facilitate the comparability between results from different studies by comparing our data with available TL data from the National Health and Nutrition Examination Survey (NHANES).

## 2. Materials and Methods

### 2.1. Study Participants

We used TL measurements, by both qPCR and flow FISH assays, from 181 healthy donors, using data we generated in the context of studies evaluating the effect of donor TL on survival after hematopoietic stem cell transplantation [15,16]. Samples were provided by the National Marrow Donor Program^®^ (NMDP, Minneapolis, MN, USA)/Center for International Blood and Marrow Transplant Research^®^ (CIBMTR) (Minneapolis, MN, USA) biorepository. The median age of study participants was 36 years (range = 19–53). 

From the NHANES database (survey cycles 1999–2000 and 2001–2002) [24], we identified 1810 adults with qPCR TL data who were 1:10 frequency-matched to our study participants by age (in 5-year categories) and sex.

### 2.2. Telomere Length Measurement 

For qPCR TL measurements, genomic DNA was extracted from peripheral blood mononuclear cells using a QIAamp DNA Blood Maxi Kit (QIAGEN, Inc., Germantown, MD, USA) for the NMDP cohort, and from whole blood using the Puregene (D-50 K) kit protocol (Gentra Systems, Inc., Minneapolis, MN, USA) for the NHANES samples [25]. Relative telomere length (RTL) was determined by a qPCR assay as described elsewhere [19]. Briefly, the method calculates the ratio of amplified telomere (T) signal to that of an autosomal single copy gene (S; *36B4* for NMDP samples, and human beta-globin for NHANES samples). The obtained value was then standardized using internal quality control (QC) samples. Method details for both cohorts are available elsewhere [16,26]. 

For flow FISH TL measurement in the NMDP cohort, cryopreserved peripheral blood mononuclear cell samples (PBMCs) were washed and then mixed with bovine thymocytes of known telomere length as an internal control. Samples were next denatured with formamide at 87 °C and hybridized with telomere-specific fluorescein labeled (CCCTAA)_3_ PNA probes. The DNA was counterstained with LDS751 DNA dye. Lymphocytes were distinguished by flow cytometry based on LDS751 fluorescence intensity and light scatter signals. TL in total lymphocytes was analyzed in the present study. Method details are described elsewhere [18]. 

### 2.3. Telomere Length Calculations in Kilobases 

From the NMDP cohort, we calculated the predicted TL by regressing the qPCR relative T/S ratio on the flow FISH total lymphocyte TL (kb) for the same individuals. The conversion equation is: TL = 3.571 + 4.978 × (T/S). 

The published conversion equation from NHANES was calculated based on the comparison of TRF from Southern blot analysis and T/S ratios using DNA samples from the human diploid fibroblast cell line IMR90 at different population doublings (https://wwwn.cdc.gov/Nchs/Nhanes/2001-2002/TELO_B.htm). The equation is: TL (kb) = 3.274 + 2.413 × (T/S).

### 2.4. Statistical Analysis

We used linear regression models and calculated the coefficient of determination (*R*^2^) to evaluate the strength of association between different measures of TL and between TL and age. Bland-Altman analysis was used to assess the agreement between kb of TL calculated from qPCR and that from flow FISH. The bias was estimated as the mean differences between calculated TL from qPCR and flow FISH TL. Limits of agreements (LoA), a range of acceptable differences, was defined as mean ± 1.96 × standard deviation (SD) as recommended previously [27,28]. To examine whether agreement between calculated TL from qPCR and flow FISH TL was different in the TL extremes (that of greatest clinical interest), we repeated the analysis in the following strata of flow FISH TL:TL ≤10th percentile (*n* = 19), 10th< and <90th percentile (*n* = 143), and ≥90th percentile (*n* = 19). The R package “blandr” was used for the Bland-Altman analysis [29].

To test whether the use of conversion equations is a valid tool for comparing results across studies, we used (1) Wilcoxon rank-sum test to compare the distribution of calculated TL (kb) in the NMDP cohort and in a sub-population from NHANES who were frequency-matched to the NMDP cohort by age and sex; and (2) linear regression models of TL on age to calculate the average annual TL attrition rate using cross-sectional data in the NMDP cohort from both the flow FISH TL and calculated TL in kb from qPCR, and in the NHANES cohort from calculated TL using the NHANES published equation. We used unweighted methods for the analyses of NHANES data, while excluding one outlier with a calculated TL > 25 kb from the analysis. 

All tests were two-sided with statistical significance defined as *p* < 0.05. All statistical analyses were performed using SAS version 9.4 (SAS Institute Inc., Cary, NC, USA) and R software version 3.4.4 (R Foundation for Statistical Computing, Vienna, Austria). Correlation, distribution and regression plots were generated using R package “ggplot2” [30].

## 3. Results

### 3.1. Flow FISH and qPCR TL in the NMDP Cohort

**Figure 1:**
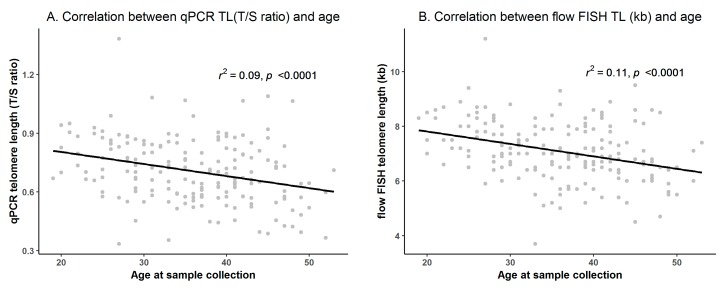
Correlation between telomere length (TL) and age (**A**) qPCR TL (T/S ratio); (**B**) Flow cytometry with fluorescence in situ hybridization (flow FISH) TL (kb).

One hundred and twelve (62%) individuals were male, and median age at blood collection was 35 years for males (range = 20–53), and 37 years for females (range = 19–52). The median relative qPCR TL (T/S ratio) was 0.7 for both males (range = 0.33–1.38) and females (range = 0.35–1.09). The median flow FISH TL was 6.9 kb for males (range = 4.7–11.2), and 7.2 for females (range = 3.7–9.5). TL was inversely correlated with age for both qPCR (*r* = −0.30, *p* < 0.0001, Figure 1A) and flow FISH (*r* = −0.33, *p* < 0.0001, Figure 1B). 

### 3.2. Comparability between Calculated kb of qPCR and Flow FISH TL in the NMDP Cohort

**Figure 2:**
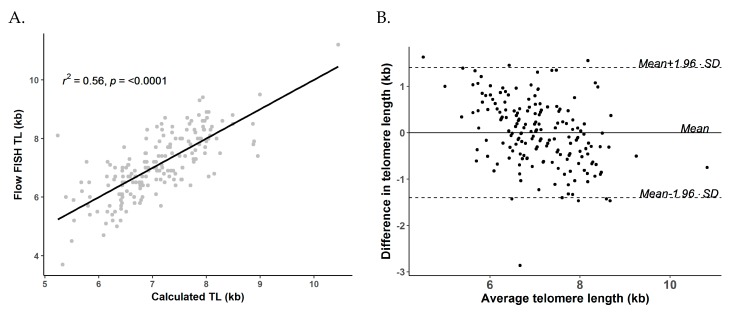
Correlation and agreement between calculated telomere length (TL) from conversion equation and flow cytometry with fluorescence in situ hybridization (flow FISH) TL in the National Marrow Donor Program^®^ (NMDP) cohort. (**A**) Correlation between calculated qPCR TL (kb) and flow FISH TL; (**B**) Bland-Altman plot of agreement between calculated TL from conversion equation and flow FISH TL.

The correlation between calculated TL from qPCR and flow FISH TL was modest (*R*^2^ = 0.56, *p* < 0.0001, Figure 2A). The median calculated TL was 7.1 kb (range = 5.2–10.5 kb). In Bland-Altman analysis, the calculated TL showed an overall agreement with flow FISH data (bias ± SD = 0.00 ± 0.72 kb, LoA = −1.40 to 1.40 kb, Figure 2B). 

**Figure 3:**
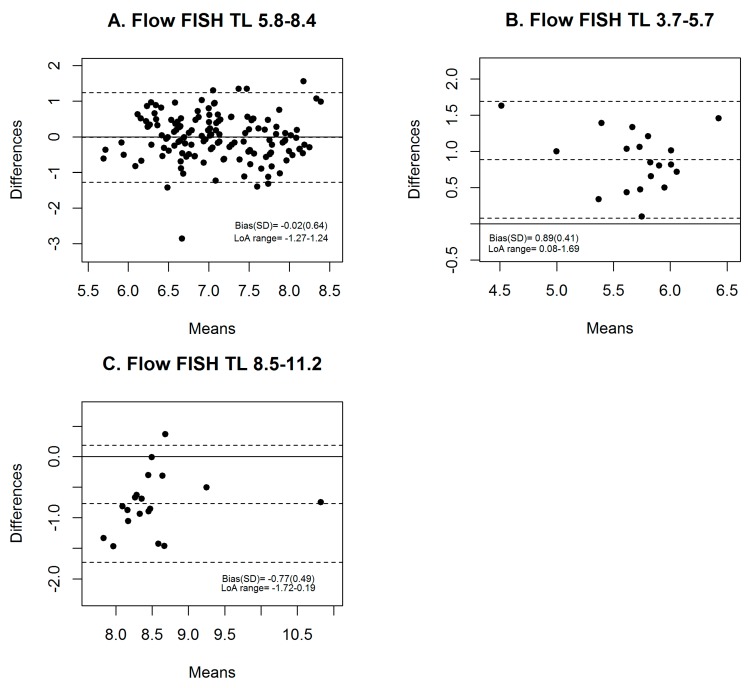
Flow cytometry with fluorescence in situ hybridization (Flow FISH) telomere length (TL) quintile stratified analysis of agreement between calculated TL from conversion equation and flow FISH TL in the National Marrow Donor Program^®^ (NMDP) cohort. (**A**) Above 10th and below 90th percentile (TL 5.8–8.4); (**B**) Flow FISH TL ≤ 10th percentile (TL 3.7–5.7); (**C**) ≥90th percentile (TL 8.5–11.2).

In analysis stratified by flow FISH TL, acceptable agreement was only observed in individuals with a flow FISH TL within the normal range (between 10th and 90th percentiles (bias = −0.02), Figure 3A). The conversion equation resulted in TL over-estimation (bias ± SD = 0.89 ± 0.41 kb, Figure 3B) in individuals with short TL (≤10th percentile) and under-estimation (bias ± SD = −0.77 ± 0.49 kb, Figure 3C) in individuals with long TL (≥90th percentile). 

### 3.3. Comparison of Calculated TL between NMDP and NHANES Cohorts

**Figure 4:**
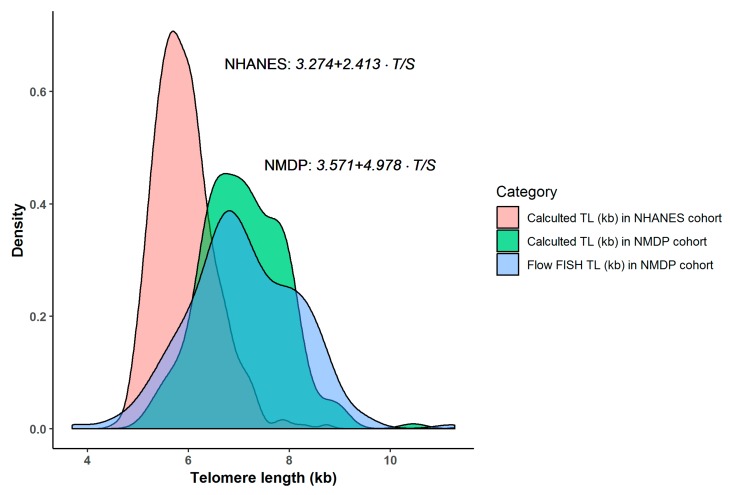
Distribution of calculated telomere length (TL) in the National Marrow Donor Program^®^ (NMDP) and National Health and Nutrition Examination Survey (NHANES) cohorts.

Using the NHANES published conversion equation, the median calculated TL in the age- and sex-matched NHANES cohort was 5.8 kb (range = 4.5–11.3 kb). The median calculated TL in NHANES and our NMDP cohort was statistically significantly different (median calculated TL = 5.8 *versus* 7.1 kb for NHANES *versus* NMDP cohort, respectively; Wilcoxon rank-sum test *p* < 0.0001). Figure 4 describes the distribution of the calculated TL in each cohort.

**Figure 5:**
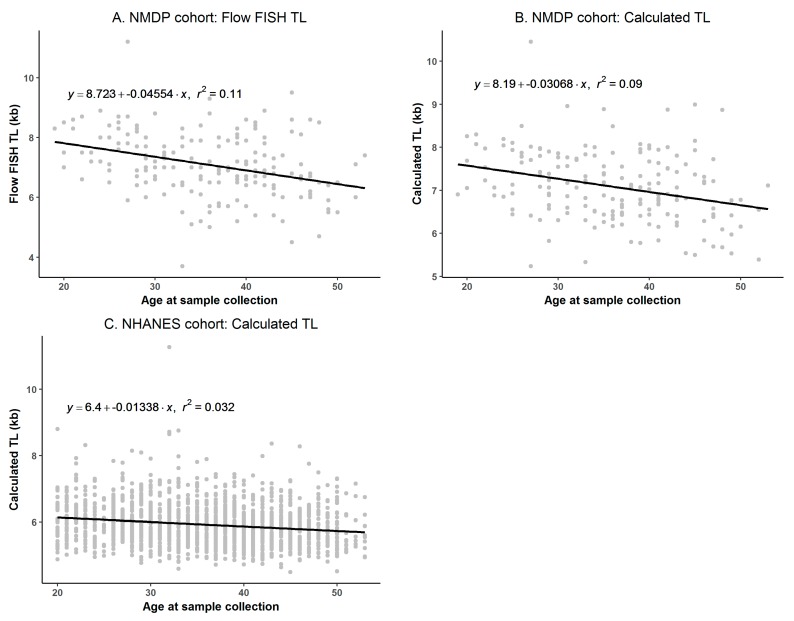
Annual telomere length (TL) attrition. (**A**) National Marrow Donor Program^®^ (NMDP) cohort: flow cytometry with fluorescence in situ hybridization (flow FISH) TL; (**B**) NMDP cohort: calculated TL using conversion equation generated from the qPCR T/S ratio; (**C**) National Health and Nutrition Examination Survey (NHANES): calculated TL using the published conversion equation.

In the cross-sectional linear regression analyses of TL on age, we observed clear differences in the estimated annual average TL attrition between both cohorts. The flow FISH TL from the NMDP cohort showed an attrition rate of 46 base pairs (bp) per year of age (Figure 5A). Using the calculated kb TL from the same individuals, a lower attrition rate was noted (31 bp/year, Figure 5B); the difference was not statistically significant (*p* = 0.22). In the NHANES cohort, the use of their published conversion equation resulted in an annual attrition rate of 13 bp/year (Figure 5C), which was significantly lower than that estimated from the calculated kb TL in the NMDP cohort (*p* = 0.02). 

## 4. Discussion

In this study, we showed that calculating the kb TL from the qPCR T/S ratio with current practice conversion equations (from linear regression models) results in imprecise estimates for individuals with short or long telomeres. Comparison of the calculated TL between our study population and age- and sex-matched individuals from the NHANES study showed that the equation for estimating the kb values of TL from the qPCR T/S ratio is only valid for the given data where the conversion equation is derived from, and cannot be used to compare TL results in different studies.

High throughput qPCR-based assays for TL measurement are being used extensively for a wide array of studies seeking to understand phenotype associations with TL. Our study and several others agree that the correlation between the qPCR TL measurement and that obtained from the highly accurate flow FISH assay is modest (*R*^2^ = 0.56 in our study, and *R*^2^ = 0.33, and 0.42 in published studies) [31,32]. Similar results were observed when comparing qPCR TL with that obtained from the Southern blot assay (*R*^2^ = 0.35 and 0.43) [31,32]. Differences in the magnitude of correlation between studies may be related to the qPCR sensitivity to assay pre-analytic factors, such as DNA extraction, and inhibitor removal methods, well position, and sample storage conditions [33,34,35,36]. A previous study showed longer RTL in samples extracted with phenol/chloroform or PureGene kit, compared with the QIAamp method [34]. A subsequent study showed about 40% shorter RTL values with magnetic-particle methods, compared to salting-out extraction methods; the difference resulted in a stronger association between RTL and cardiovascular disease with salting-out DNA extraction [35].

Our data showed that qPCR results are less reliable for those with the shortest and longest TL. This is important, particularly in clinical studies where the TL association with disease is usually at the extremes. Our previous study evaluating the ability of qPCR TL to identify patients with DC (TL usually below the 1st percentile-for-age with flow FISH assay) showed that approximately 60% of patients were misclassified by qPCR TL (TL being above the 1st percentile-for-age) [37]. In agreement with our finding, a previous study showed a higher degree of bias in the calculated kb of TL from qPCR in patients with telomere-biology disorders (including DC) than that observed in healthy individuals (average bias in the comparison with flow FISH TL = −1.15 *versus* −0.6 kb, respectively) [31]. A more sophisticated modeling strategy in a large study, with enough individuals with extreme TL values, is needed to develop a robust conversion equation.

We observed substantial differences in the distribution of calculated TL in the NMDP cohort and that of the NHANES (median = 7.1 *versus* 5.8, and ranges = 5.2–10.5 *versus* 4.5–11.3, respectively), despite the similarity in age and sex distribution. This difference, at least in part, is related to the source of the conversion equation where we used the same samples for flow FISH analysis, while a fibroblast cell line was used for the TRF analysis in NHANES. This is also observed in large studies using cell line calibrators, where the median calculated TL ranged between 3.2 and 4.1 kb [20,21,22]; values much shorter than expected. A recent study using TRF identified a leukocyte TL of 5 kb as the point defining critically short TL; the study included more than 10,000 adults, almost none of those younger than age 60 had a TL <5 kb [38]. 

In the current study, our flow FISH data showed a TL attrition rate of 46 bp/year. This is similar to the rate observed in an independent cohort of more than 800 healthy individuals with flow FISH TL measured at the same laboratory (lymphocyte TL annual attrition rate in adults = 43 bp) [39], and a second cohort of 192 individuals measured in another laboratory [40]. Another study including 154 healthy individuals showed an annual TL attrition of 64 bp for TRF and 50 bp for flow FISH [41]. The observed difference in TL attrition rate from conversion equations in different cohorts of similar age and sex (31 bp/year in the NMDP samples and 13 bp/year in the NHANES population) argues against the usability of TL conversion equations in comparing results across studies. 

Our study is limited by the unavailability of a direct absolute TL measurement (e.g., results from TRF) for comparison in the NMDP cohort. Instead, we used a flow FISH TL measurement that depends on TRF calibration via a reference sample. Yet, flow FISH is known for its accuracy and the strong correlation with TL measured by TRF [32,42]. This assures the validity of our results. 

In conclusion, our findings demonstrate that the use of linear equations for converting the qPCR T/S ratio to kb TL may introduce bias particularly at the bottom and top percentiles of TL. Caution should be exercised when comparing results from different studies using calculated TL from qPCR T/S ratio data since values are affected by the possible inter-laboratory variability in TL qPCR protocols and the underlying calibrator used.
